# Global economic burden per episode for multiple diseases caused by group A *Streptococcus*

**DOI:** 10.1038/s41541-023-00659-1

**Published:** 2023-05-15

**Authors:** Jung-Seok Lee, Sol Kim, Jean-Louis Excler, Jerome H. Kim, Vittal Mogasale

**Affiliations:** 1 International Vaccine Institute, Seoul, South Korea; 2 College of Natural Sciences, Seoul National University, Seoul, South Korea

**Keywords:** Public health, Bacterial infection

## Abstract

Considering the lack of existing evidence on economic burden for diseases caused by group A *Streptococcus*, we estimated the economic burden per episode for selected diseases. Each cost component of direct medical costs (DMCs), direct non-medical costs (DNMCs), and indirect costs (ICs) was separately extrapolated and aggregated to estimate the economic burden per episode by income group as classified by the World Bank. Adjustment factors for DMC and DNMC were generated to overcome related data insufficiencies. To address uncertainty surrounding input parameters, a probabilistic multivariate sensitivity was carried out. The average economic burden per episode ranged from $22 to $392 for pharyngitis, $25 to $2,903 for impetigo, $47 to $2,725 for cellulitis, $662 to $34,330 for invasive and toxin-mediated infections, $231 to $6,332 for acute rheumatic fever (ARF), $449 to $11,717 for rheumatic heart disease (RHD), and $949 to $39,560 for severe RHD across income groups. The economic burden for multiple Group A *Streptococcus* diseases underscores an urgent need to develop effective prevention strategies including vaccines.

## Introduction

Group A *Streptococcus* (GAS) causes a wide spectrum of diseases, from pharyngitis and superficial skin infections to more severe diseases such as invasive infections, immune-mediated disease such as acute rheumatic fever (ARF) and direct sequelae of immune-mediated disease such as rheumatic heart disease (RHD)^1–3^. The global health and economic burden of GAS infections is disproportionately concentrated in low- and middle-income countries, as well as Pacific and Indigenous populations of Australia and New Zealand, and of Canada and Native American/Alaskan Native populations of the United States^2,4,5^. More specifically, the prevalence of RHD among children aged 5 to 14 years is highest in sub-Saharan Africa at 5.7 per 1000 children, followed by the Pacific and Indigenous populations of Australia and New Zealand (3.5 per 1000), and southcentral Asia (2.2 per 1000)^6^. Among the severe GAS diseases, RHD was identified as the cause of the greatest number of deaths due to GAS, and the estimated burden of RHD was updated in 2015 showing 319,400 deaths^7^. Considering there are over 111 million cases of GAS pyoderma and 616 million cases of GAS pharyngitis each year^4^, the disease and economic burden of GAS in total would be significant.

The diseases caused by GAS can be categorized into five disease categories: (1) superficial, (2) locally invasive, (3) invasive and toxin-mediated, (4) immune-mediated, and (5) direct sequelae of immune-mediated. There are more than 20 syndromes which fall into those categories^8^, including pharyngitis and impetigo (superficial), tonsillitis and cellulitis (locally invasive), scarlet fever, streptococcal toxic shock syndrome (toxin-mediated), puerperal sepsis, bacteremia, pneumonia, and necrotizing fasciitis (invasive), ARF and acute poststreptococcal glomerulonephritis (APSGN) (immune-mediated), and RHD and chronic kidney disease (direct sequelae of immune-mediated). Although immunology and genetics of GAS-associated diseases are complex^6,9^, there is a clear sequential effect of the bacterium over the course of diseases. As an example, if seemingly benign GAS pharyngitis is not treated in a timely and proper manner, the infection can further develop into ARF^10^, which results into heavier health and economic burden^11^. The onset of ARF is typically 2 to 4 weeks after GAS pharyngitis^12^ and often recurs, causing heart damage^6^. Due to the linkage identified between the GAS pharyngitis and ARF, countries concerned about preventing ARF recommend that Strep throat infections are treated with an appropriate antibiotic regimen such as the oral antibiotic regimen (Penicillin V or amoxicillin for 10 days), or intramuscular benzathine penicillin G injection as prophylaxis^12,13^. Likewise, disease progression from ARF to RHD indicates progressive valvular heart disease (VHD) represented by mitral stenosis and mitral regurgitation^6,14,15^, which eventually leads to permanent heart damage and death^16^. It was estimated that carditis progresses to chronic RHD in 60% of ARF cases^4,17^.

Considering that GAS causes a wide range of disease syndromes including immune-mediated sequelae, the economic burden of GAS may be substantial. However, little attention has been paid to the estimation of economic burden for GAS infections particularly in lower-middle-income and low-income countries. With multiple diseases caused by GAS and the lack of existing evidence on economic burden estimations, it becomes highly challenging to estimate the economic burden of GAS infections at the global level (by country income group or level as classified by the World Bank). The limited number of existing data is still a major hurdle even when estimating the economic burden of ARF or RHD which has been more frequently mentioned in the context of GAS-associated diseases than other manifestations such as skin infections, or invasive infections. The current study is carried out as part of the Strep A Vaccine Global Consortium (SAVAC) which aims at generating evidence useful for vaccine development and policy decision-making at the global- and national-levels (https://savac.ivi.int/). While the scarcity of existing information on the economic burden of GAS infections remains challenges, the main interest of the current analysis lies in estimating the average economic burden per episode for selected diseases caused by GAS where at least the minimum level of observed data exists.

## Results

### Input values

Input parameters such as DMC adjustment factors, DNMC adjustment factors, and days of productivity losses are summarized in Table 1. A majority of DMC adjustment factors for pharyngitis, skin infections, and ARF were lower than 1 indicating that the observed DMC values were less than the crude DMC estimated based on the WHO unit costs. On the other hand, this was the opposite for ITMI, RHD, and severe RHD showing most DMC adjustment factors being greater than 1. As expected, the DNMC adjustment factors only took up a small proportion of GDP per capita across income groups and diseases. The duration of productivity loss was much lower for pharyngitis and skin infections than for other disease categories. While productivity loss days were slightly higher for RHD than for severe RHD, this was mainly due to the large variance in the reported sick days for RHD over a 10-year period compared to the relatively consistent duration of illness for severe RHD.

**Table 1:** Input parameters.

Group	Input parameter	Pharyngitis			Impetigo			Cellulitis			Invasive and toxin-mediated infections		
		Mode	Min	Max	Mode	Min	Max	Mode	Min	Max	Mode	Min	Max
HIC	DMC adjustment factor	0.6993	0.1073	1.6802	1.5284	1.2227	1.8341	0.5024	0.4019	0.6029	2.4046	0.6837	4.1380
	DNMC adjustment factor	0.0002	0.0001	0.0003	0.0002	0.0001	0.0002	0.0004	0.0003	0.0005	0.0263	0.0001	0.0670
	Days of productivity loss	5	3	7	5	2	19	10	2	16	21	17	25
UMIC	DMC adjustment factor	0.4275	0.1878	0.9669	1.5284	1.2227	1.8341	0.1426	0.1141	0.1711	2.4046	0.6837	4.1380
	DNMC adjustment factor	0.0002	0.0001	0.0003	0.0002	0.0001	0.0002	0.0004	0.0003	0.0005	0.0263	0.0001	0.0670
	Days of productivity loss	5	3	7	5	2	19	10	2	16	21	17	25
LMIC	DMC adjustment factor	0.5135	0.4108	0.6162	0.0354	0.0284	0.0425	0.1426	0.1141	0.1711	2.4046	0.6837	4.1380
	DNMC adjustment factor	0.0004	0.0003	0.0004	0.0003	0.0003	0.0004	0.0007	0.0006	0.0009	0.0055	0.0044	0.0066
	Days of productivity loss	5	3	7	5	2	19	10	2	16	21	17	25
LI	DMC adjustment factor	0.5135	0.4108	0.6162	0.0354	0.0284	0.0425	0.1426	0.1141	0.1711	2.4046	0.6837	4.1380
	DNMC adjustment factor	0.0004	0.0003	0.0004	0.0003	0.0003	0.0004	0.0007	0.0006	0.0009	0.0055	0.0044	0.0066
	Days of productivity loss	5	3	7	5	2	19	10	2	16	21	17	25

Group	Input parameter	ARF			RHD			Severe RHD		
		Mode	Min	Max	Mode	Min	Max	Mode	Min	Max
HIC	DMC adjustment factor	0.4663	0.0132	1.1217	0.5736	0.0266	1.3098	2.8952	1.0746	5.0017
	DNMC adjustment factor	0.0005	0.0004	0.0006	0.0294	0.0001	0.0748	0.0301	0.0043	0.0558
	Days of productivity loss	21	12	35	23	7	59	21	20	22
UMIC	DMC adjustment factor	2.3189	1.8573	2.6127	1.5726	1.3389	1.9422	6.5811	5.5131	7.2603
	DNMC adjustment factor	0.0005	0.0004	0.0006	0.0294	0.0001	0.0748	0.0301	0.0043	0.0558
	Days of productivity loss	21	12	35	23	7	59	21	20	22
LMIC	DMC adjustment factor	0.7921	0.6337	0.9505	1.3145	0.5138	1.9261	3.7741	0.8579	6.0140
	DNMC adjustment factor	0.0005	0.0004	0.0006	0.0062	0.0049	0.0074	0.0301	0.0043	0.0558
	Days of productivity loss	21	12	35	23	7	59	21	20	22
LI	DMC adjustment factor	0.7921	0.6337	0.9505	1.3145	0.5138	1.9261	3.7741	0.8579	6.0140
	DNMC adjustment factor	0.0005	0.0004	0.0006	0.0062	0.0049	0.0074	0.0301	0.0043	0.0558
	Days of productivity loss	21	12	35	23	7	59	21	20	22

### Economic burden per episode

Table 2 shows the economic burden per episode for the diseases. The estimated economic burden ranged from $22 to $392 for pharyngitis, $25 to $2,903 for impetigo, $47 to $2,725 for cellulitis, $662 to $34,330 for ITMI, $231 to $6,332 for ARF, $449 to $11,717 for RHD, and $949 to $39,560 for severe RHD. The economic burden per episode was the highest for those who experienced severe RHD or ITMI and the lowest for patients with pharyngitis. In general, the economic burden was greater in higher income groups than lower income groups across the seven diseases when costs were expressed in US dollars (based on official market exchange rates). However, it is interesting to observe that the economic burden of RHD and severe RHD in the upper-middle-income group became similar to the economic burden of RHD and severe RHD in the high-income group when the costs were converted into international dollars using the Purchasing Power Parities (PPP) conversion factor (Table 3). Moreover, the cost of illness for ARF in the upper-middle income group surpassed the cost for ARF in the high-income group as expressed in international dollars. In other words, when following the law of one price reflecting people’s living standards comparably across countries, the economic burden of ARF, RHD, or severe RHD in the upper-middle-income group would be similar to or greater than those estimated for the high-income group.

**Table 2:** Average economic burden per episode in US$.

Disease	HIC			UMIC			LMIC			LIC		
	Mean	LCI 95%	HCI 95%	Mean	LCI 95%	HCI 95%	Mean	LCI 95%	HCI 95%	Mean	LCI 95%	HCI 95%
Pharyngitis	$392.0	$283.8	$501.2	$82.8	$62.4	$104.6	$48.9	$36.7	$60.6	$21.8	$16.6	$27.0
Impetigo	$2,902.7	$2,429.1	$3,417.4	$633.6	$528.9	$748.2	$57.0	$21.1	$111.8	$24.8	$9.1	$48.5
Cellulitis	$2,725.1	$2,275.5	$3,148.2	$254.7	$186.0	$316.0	$111.9	$69.0	$150.6	$46.9	$27.9	$64.0
ITMI	$34,330.3	$17,808.7	$50,467.0	$7,301.4	$3,832.9	$10,767.7	$1,788.8	$970.9	$2,603.2	$661.6	$372.5	$954.6
ARF	$6,332.2	$2,535.2	$10,640.3	$5,276.5	$4,649.3	$5,818.6	$591.6	$504.3	$683.5	$230.7	$194.5	$268.9
RHD	$11,716.9	$5,122.0	$18,868.4	$5,620.7	$4,905.0	$6,393.2	$1,180.4	$778.4	$1,580.8	$448.5	$292.7	$598.3
sRHD	$39,560.1	$22,487.6	$57,338.5	$18,089.4	$16,310.9	$19,614.7	$2,612.1	$1,389.2	$3,715.1	$948.8	$503.4	$1,353.5

*LCI* 95% lower bound of the 95% confidence interval, *HCI* higher bound of the 95% confidence interval.

**Table 3:** Average economic burden per episode in I$.

Disease	HIC			UMIC			LMIC			LIC		
	Mean	LCI 95%	HCI 95%	Mean	LCI 95%	HCI 95%	Mean	LCI 95%	HCI 95%	Mean	LCI 95%	HCI 95%
Pharyngitis	$457.8	$331.6	$584.7	$174.6	$131.2	$220.3	$122.3	$92.3	$151.2	$60.9	$46.7	$75.1
Impetigo	$3,267.5	$2,730.3	$3,860.2	$1,459.6	$1,225.6	$1,712.5	$141.1	$53.2	$275.8	$67.8	$25.4	$132.0
Cellulitis	$3,076.7	$2,560.5	$3,561.0	$560.1	$418.7	$687.7	$286.9	$181.4	$382.2	$134.1	$82.3	$180.6
ITMI	$38,447.6	$19,987.3	$56,455.5	$16,996.6	$8,863.3	$25,112.7	$5,010.4	$2,688.1	$7,327.4	$2,144.4	$1,186.8	$3,115.6
ARF	$7,146.6	$2,903.9	$11,968.6	$12,283.0	$10,820.2	$13,548.3	$1,608.9	$1,376.8	$1,850.7	$715.1	$608.1	$826.9
RHD	$13,220.5	$5,820.8	$21,220.0	$13,029.2	$11,396.3	$14,803.5	$3,263.6	$2,134.3	$4,380.4	$1,425.0	$923.9	$1,895.1
sRHD	$44,306.3	$25,235.5	$64,170.3	$42,275.2	$38,100.4	$45,833.8	$7,326.5	$3,856.4	$10,458.5	$3,084.2	$1,605.7	$4,421.5

*LCI* 95% lower bound of the 95% confidence interval, *HCI* higher bound of the 95% confidence interval.

**Fig. 1:**
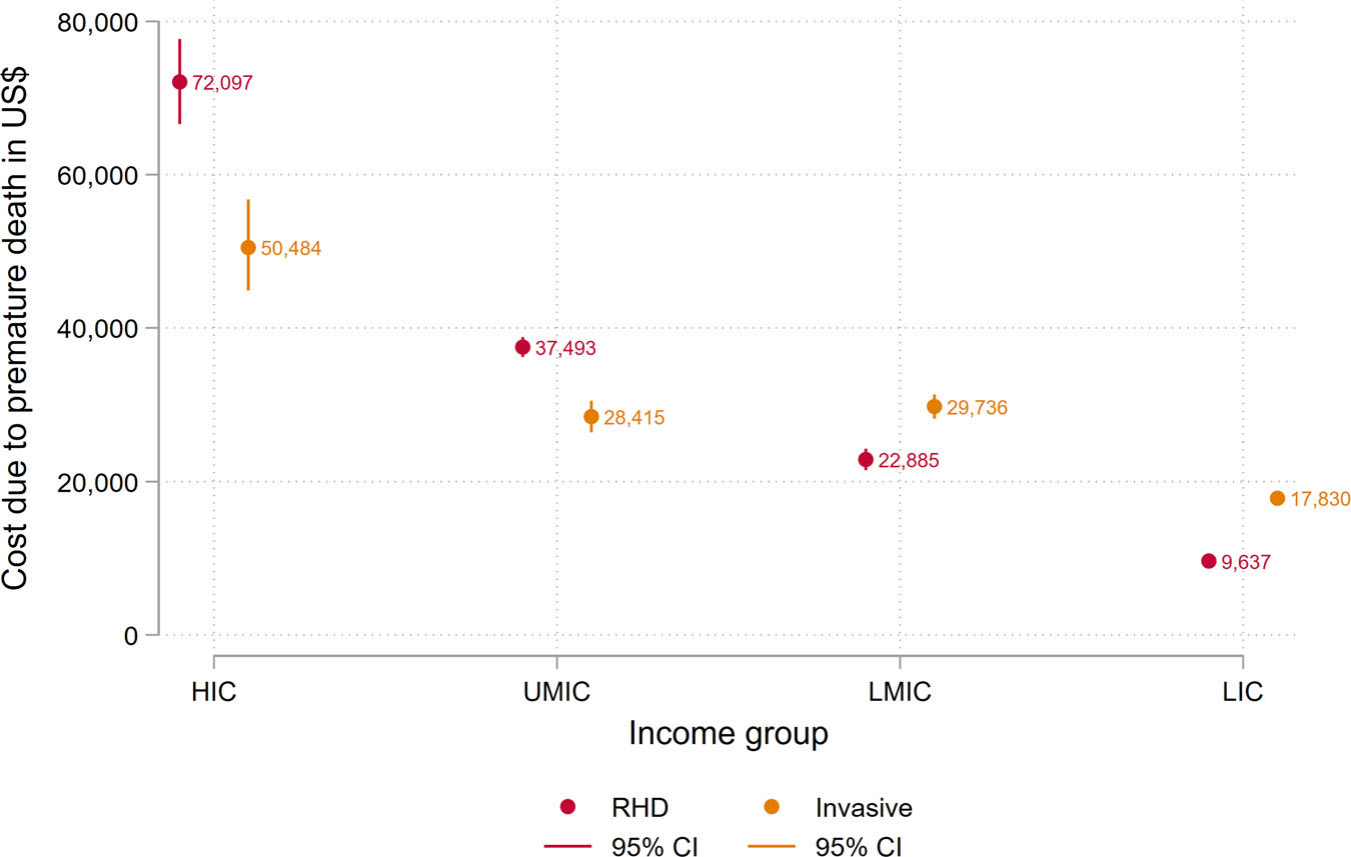
Productivity loss due to premature death from RHD and ITMI. Productivity losses due to premature death from RHD and ITMI caused by group A *Streptococcus* are presented by income group in the graph above, with the 95% confidence intervals (CIs). HIC High Income Countries, UMIC Upper-Middle Income Countries, LMIC Lower-Middle Income Countries, and LIC Low Income Countries.

Productivity loss due to premature death from RHD and invasive infections is shown in Fig. 1. While productive years lost were the lowest in the high-income group given the weighted average age of death being the highest, the cost due to early death was the greatest in the high-income group and the lowest in the low-income group. This is mainly because patients in the high-income group are expected to earn more than those in lower-income groups.

**Fig. 2:**
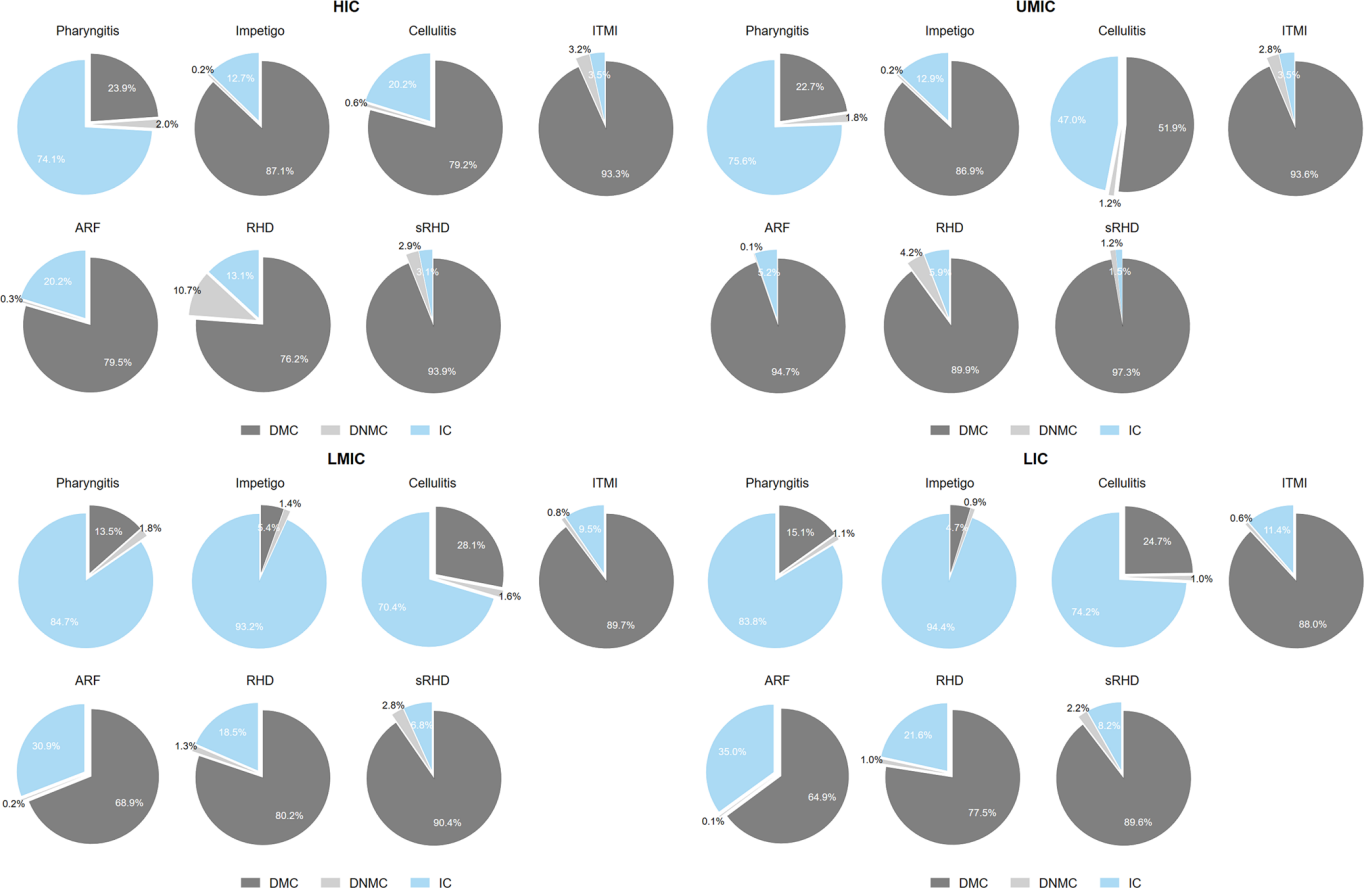
Proportion of economic burden by cost type, stratified by income group and group A streptococcal syndrome. HIC High Income Countries, UMIC Upper-Middle Income Countries, LMIC Lower-Middle Income Countries, LIC Low Income Countries, DMC Direct Medical Cost, DNMC Direct Non-Medical Cost, IC Indirect Cost, ARF Acute Rheumatic Fever, RHD Rheumatic Heart Disease, sRHD severe Rheumatic Heart Disease.

Figure 2 illustrates the proportions of economic burden by cost type per GAS syndrome. For patients with pharyngitis, IC accounts for the biggest proportion out of the total economic burden followed by DMC and DNMC in all four income settings. On the other hand, the proportion of DMC increases significantly as moving towards ARF, severe RHD, and ITMI. In particular, the proportion of DMC becomes the dominant cost factor compared to IC and DNMC for those who develop invasive GAS infections or severe RHD. This is because treatment costs required for pharyngitis are relatively smaller than treatment costs associated with more severe forms of GAS infection such as severe RHD. In case of skin infections, while DMC is the main cost driver in higher income groups (HIC, UMIC), IC appears to be the dominant factor in lower income groups (LMIC, LIC). Overall, the DNMC proportions are the least across all diseases and income groups, and the proportion of IC appears to be greater in lower income groups (LMIC, LIC) than in higher income groups (HIC, UMIC) although the differences are marginal.

**Fig. 3:**
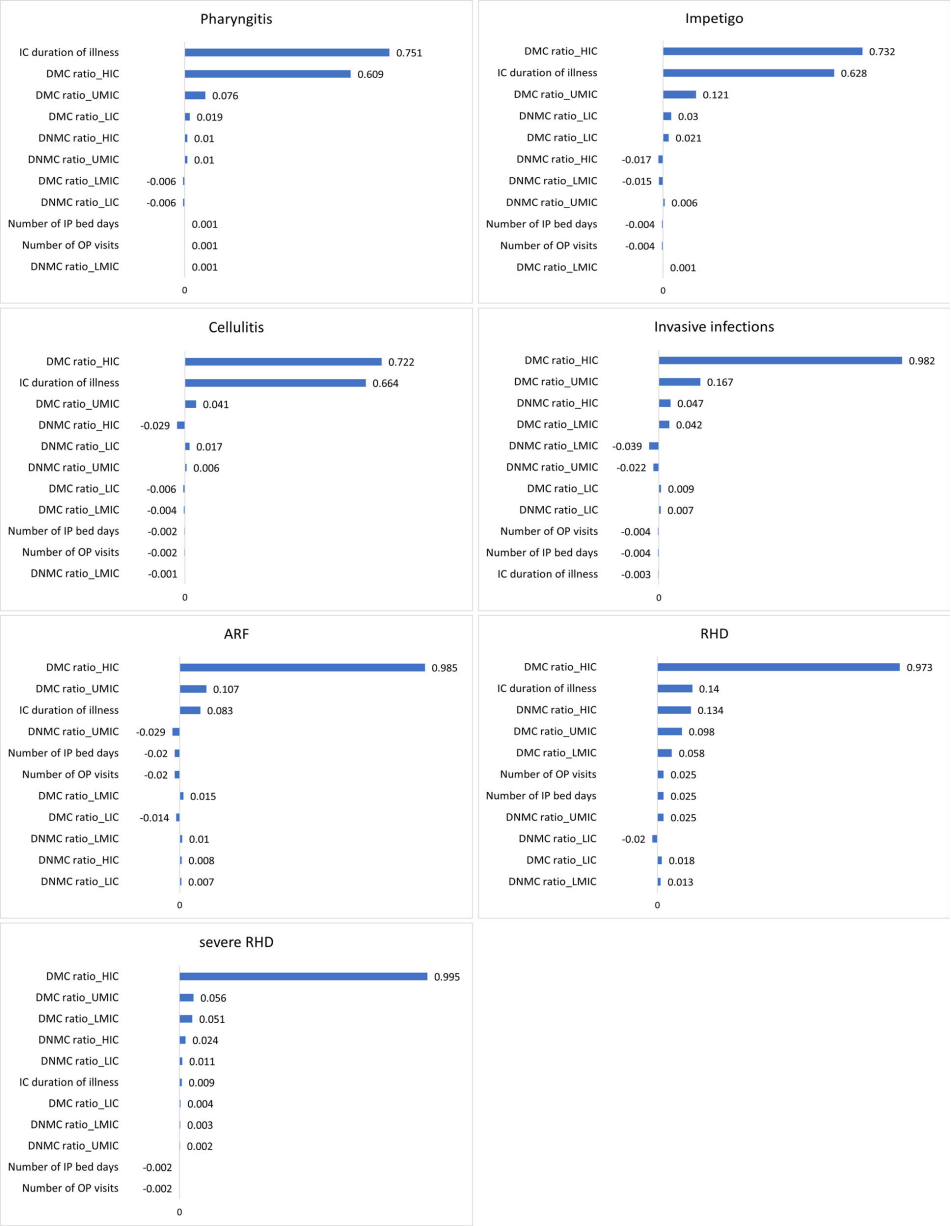
Spearman’s rank correlation coefficients. The tornado plots are presented based on Spearman’s rank correlation coefficients for all diseases. Input parameters: duration of illness, DMC adjustment factors, DNMC adjustment factors, number of inpatient bed-days, and number of outpatient visits.

The probabilistic multivariate sensitivity analysis is shown in Fig. 3. The duration of illness had the highest impact on the overall economic burden per episode for pharyngitis followed by the DMC adjustment factors in the high- and upper-middle income settings. The DMC adjustment factors in the high-income settings appeared to be the most sensitive indicator to the results for the rest of the diseases. While the DMC adjustment factors in three income settings were predominant factors for the economic burden of severe RHD, the duration of illness and the DNMC adjustment factor in the high-income settings had some level of impacts on the economic burden of RHD and ARF following the DMC adjustment factors in the high-income settings. In addition, the duration of illness appeared to be an important indicator affecting the economic burden of impetigo and cellulitis.

## Discussion

The current study estimated the economic burden per episode for GAS pharyngitis, impetigo, cellulitis, ITMI, ARF, RHD, and severe RHD, based on existing studies. The estimation of economic burden based on existing studies was previously conducted for other diseases as well^18,19^. Overall, existing studies which reported cost of illness for GAS-induced diseases were scarce. In particular, there was no study available from low-income countries. Given the insufficient number of observed data points, the adjustment factors for DMC were generated by comparing the WHO-CHOICE data with the empirical DMC costs identified in existing studies by income group. The adjustment factors and the duration of illness by disease category were then used to extrapolate economic burden of the seven diseases by income group.

The estimated economic burden per episode for each of the seven diseases was greater in the high-income group than in lower income groups. The ranges of economic burden per episode by each syndrome were wide from low-income countries to high-income countries. This is mainly due to observed values being largely variable across income groups. It should be noted that when economic burden was expressed in international dollars, the variations in economic burden among some income groups appeared to be different compared to the variations observed based on market exchange rates. In other words, when accounting for the differences in the cost of living, the economic burden of ARF, RHD, and severe RHD in the upper-middle income group became similar to or greater than the ones of the high-income group. The economic burden of more severe forms of chronic illnesses such as RHD and severe RHD far outweighed the costs for GAS pharyngitis. Especially, the economic burden of severe RHD appeared to be similar to the costs incurred due to cancer: $42,111 (€35,518) per cervical cancer episode in Norway^20^, $8,066-$22,888 per cervical cancer episode in China^21^, and $1340–10,914 per breast cancer (stage I/II) in Kenya^22^.

Some areas of uncertainty deserve attention. First, the average number of outpatient visits and the duration of hospital stays for each disease category were estimated based on A SINGLE database published by the Health Insurance Review & Assessment Services OF SOUTH KOREA. It should be noted that the number of outpatient visits or the length of hospital stays may differ in other income settings, and the use of the single data source may not fully account for differences by country. Nonetheless, identifying a standardized duration of illness for each of multiple GAS infections by different income settings is highly challenging due to varying degrees of subgroup definitions and categories for each disease syndrome. The use of the healthcare big data hub system provided the detailed list of standardized sub-categories within each of the diseases, and such information is often absent or non-standardized in the existing literature. A 10-year period of longitudinal data was evaluated for each of the disease subgroups in order to capture any variations which had occurred due to various reasons including medical advances over time, and this may, to some extent, reflect the differences in other income groups. In addition, the variations captured during a 10-year period were further taken into account in the sensitivity analysis. Second, it would have been more accurate if each cost were reported by age group. However, there were only a few studies which broadly categorized costs into two age cohorts (i.e., pediatric vs. adult) for the selected diseases caused by GAS^8,23,24^. Since cost data by age group were very limited, a small number of cost data points reported by pediatric and adult were averaged. Third, some of the DMC and DNMC adjustment factors particularly in the low-income group were not directly estimated due to the absence of data, thus the adjustment factors from similar income groups were applied. However, extensive sensitivity analyses were carried out with a Monte Carlo simulation, and all the results were presented with the 95% confidence intervals. Fourth, some studies did not clearly indicate the duration of illness per episode in their estimates. Given the insufficient number of existing data, we still included such studies even if there was no exact duration indicated. Assuming that a patient would not experience recurrent episodes (i.e., RHD) within the same year, the cost was then considered to be the cost per episode per year. Fifth, it is known that the use of antibiotics such as intramuscular penicillin is effective in reducing the disease progression but also causes allergic reactions which may contribute to additional treatment costs. However, these potential costs were not considered in the current study as there were only a few studies which reported the amount of additional costs caused by allergic reactions. Sixth, given diverse subgroups of diseases caused by GAS, it was challenging to calculate a standardized duration of illness for each disease. The current study utilized the number of outpatient visits and the days of hospitalization as a proxy for the duration of illness for the diseases except pharyngitis. However, it should be noted that our IC values might be conservative if one assumed that the duration of illness would be longer than the period of health facility visits. Lastly, given that cellulitis is caused by multiple pathogens, and the estimated proportion of all cellulitis is not known, existing estimates range widely [Jeffrey Cannon et al., “Modalities of group A streptococcal prevention and treatment and their economic justification”, submitted to the current special issue, npj Vaccines].

While WHO prioritized GAS vaccine development in 2014, several vaccine candidates are still at their early stage of the development^25^. Considering a significant amount of economic burden for multiple diseases caused by GAS, it is critical to fill existing gaps in primary data sources on the economic burden of diseases caused by GAS, paying special attention to the lack of evidence in low-income countries. In addition, there is an urgent need to develop new prevention strategies including vaccines. Missing an opportunity to treat mild GAS infections such as sore throat may not only exacerbate the symptoms resulting in more severe illnesses but facilitate the spread of the disease through host-to-host transmission. Thus, preventing benign GAS infections (i.e., by vaccination) is key to reduce the disease burden of diverse types of GAS-induced infections, as well as the economic burden of autoimmune diseases or its sequelae.

## Methods

Considering the number of studies available by disease, the following diseases were selected: pharyngitis, impetigo, cellulitis, invasive and toxin-mediated infections (ITMI), ARF, RHD, and severe RHD. In the current study, severe RHD was defined as RHD patients who experienced heart failures or had to undergo surgical procedures. The initial literature search was conducted by another consortium partner, the Telethon Kids Institute (TKI), Perth Australia. Each cost component of direct medical cost (DMC), direct non-medical cost (DNMC), and indirect cost (IC) was separately extrapolated and aggregated to estimate the average economic burden per episode for each disease by income group as classified by the World Bank. Because the current study extrapolates economic burden for each cost components (DMC, DNMC, and IC) separately, studies which reported the combined cost among any combination of DMC, DNMC, and IC, were excluded. In the literature, costs were collected over varying time periods and expressed in different currency units, thus crude costs were first inflated to year 2018 using GDP deflator values and converted into USD based on official exchange rates provided by the World Bank^26^.

### Direct medical costs

In order to estimate direct medical costs (DMC), the DMC values identified through the literature search were first compared with the WHO-CHOICE (CHOosing Interventions that are Cost-Effective) unit cost database. DMC included fees associated with consultations (GP visits), medications, laboratory tests (diagnostics), inpatient care (hospitalization), surgery, and all other medical examinations (i.e., X-ray, echocardiogram, etc.). The WHO-CHOICE database contained the cost of hospital bed-days, as well as of outpatient visits at different types of facilities^27^: health centres with or without beds, primary-level, secondary-level, and teaching (tertiary-level) hospitals. Understanding that multiple diseases induced by GAS accompany varying severities of symptoms requiring different levels of medical treatment, it was assumed that patients would visit one of the three health facility levels and receive treatment, depending on a type of disease that they had contracted. For example, as shown in Table 4, patients with pharyngitis (excluding those who undergo surgical procedures, i.e., tonsillectomy) were assumed to visit primary hospitals as outpatient. On the other hand, patients who develop more severe illnesses such as RHD, or severe RHD would likely receive treatment at more advanced health facilities (i.e., secondary- or tertiary-level hospitals) requiring both hospitalization and outpatient visits (pre- or post-hospitalization).

**Table 4:** Health facility level and visit type by disease.

Disease	Health facility level	Visit type
Pharyngitis	Primary	Outpatient
Impetigo	Primary	Outpatient, Inpatient
Cellulitis	Primary	Outpatient, Inpatient
Invasive and toxin-mediated infections	Tertiary	Outpatient, Inpatient
Acute rheumatic fever (ARF)	Secondary	Outpatient, Inpatient
Rheumatic heart disease (RHD)	Secondary	Outpatient, Inpatient
Severe RHD	Tertiary	Outpatient, Inpatient

In addition to health facility levels, the number of outpatient visits and the duration of hospital stay were taken into account for each disease. The Healthcare big data hub system published by the Health Insurance Review & Assessment Services in South Korea^28^ was accessed to extract the following indicators: (1) the frequency of visits, (2) the duration of bed-days, and (3) the number of inpatients (or outpatients) who received medical treatment due to each of the seven diseases listed in Table 4. Table 5 shows the details of subgroups which were categorized into each of the diseases to estimate the average number of outpatient visits and the duration of hospitalization. For example, the values for the number of outpatient visits and the duration of hospital stays were extracted from the Streptococcal meningitis subgroup under the bacterial meningitis group. To understand the variability of the number of outpatient visits or inpatient bed-days over time, the datasets were evaluated for a 10-year period from 2010 to 2019 (see supplementary Table 2). The number of outpatient visits per episode was estimated by dividing the total number of outpatient visits by the total number of outpatients. Similarly, the number of hospital bed-days per episode was calculated by dividing the total number of hospital bed-days for the syndrome of interest by the total number of inpatients who were diagnosed with the syndrome of interest. The mean of a 10-year period for each disease was used as an average number of outpatient visits (or inpatient bed-days) per episode.

**Table 5:** Specific disease syndromes included in each of the 7 key GAS syndromes.

Disease	General description	Subgroup	GAS-specificity
GAS pharyngitis	Streptococcal pharyngitis (J02.0)	-	GAS-specific
Impetigo (L01)	Bacterial infection of superficial skin	-	Not GAS-specific. This diagnosis includes conditions that are caused by group A *Streptococcus* and *Staphylococcus aureus*.
Cellulitis (L03)	Bacterial infection of dermis and subcutaneous tissue	-	Not GAS-specific. This diagnosis includes conditions that are caused by group A *Streptococcus*, *Streptococcus pneumoniae*, and *Staphylococcus aureus*.
Invasive and toxin-mediated infection	Streptococcal sepsis (A40)	Septicemia due to streptococcus, group A (A40.0)	GAS-specific
	Scarlet fever (A38)	-	GAS-specific
	Fibroblastic disorders (M72)	Necrotizing fasciitis (M72.6)	GAS-specific
	Other bacterial diseases, not elsewhere classified (A48)	Toxic shock syndrome (A48.3)	Not GAS-specific. This diagnosis includes conditions that are caused by group A *Streptococcus* and *Staphylococcus aureus*.
	Pyogenic arthritis (M00)	Other streptococcal arthritis and polyarthritis (M00.2)	Not GAS-specific. This diagnosis includes conditions that are caused by *Streptococcus* species and *Staphylococcus aureus*^36–38^.
	Bacterial meningitis, not elsewhere classified (G00)	Streptococcal meningitis (G00.2)	Not GAS-specific. This diagnosis includes conditions that are caused by *Streptococcus* species.
	Bacterial pneumonia, not elsewhere classified (J15)	Pneumonia due to other streptococci (J15.4)	Not GAS-specific. This diagnosis includes conditions that are caused by *Streptococcus* species other than group B *Streptococcus* and *Streptococcus pneumoniae*.
	Acute and subacute endocarditis (I33)	Acute and subacute infective endocarditis (I33.0)	Not GAS-specific. This diagnosis includes acute and subacute endocarditis that are bacterial, infective not otherwise specified, lenta, malignant, septic, ulcerative. Causative pathogens are hence diverse.
	Puerperal sepsis (O85)	-	Not GAS-specific. This diagnosis includes puerperal endometritis, fever, peritonitis, sepsis. Causative pathogens are hence diverse.
	Osteomyelitis (M86)	-	Not GAS-specific. This diagnosis represents any osteomyelitis, excluding osteomyelitis due to *Salmonella*, osteomyelitis of jaw, and osteomyelitis of vertebra. Causative pathogens are hence diverse.
Acute Rheumatic Fever	Rheumatic fever without mention of heart involvement (I00)	-	GAS-specific
Rheumatic Heart Disease	Rheumatic fever with mention of heart involvement (I01)	Acute rheumatic pericarditis (I01.0)	GAS-specific.
		Acute rheumatic endocarditis (I01.1)	
		Acute rheumatic myocarditis (I01.2)	
		Other acute rheumatic heart disease (I01.8)	
		Acute rheumatic heart disease, unspecified (I01.9)	
Severe RHD	Rheumatic mitral valve diseases (I05)	Mitral stenosis (I05.0)	GAS-specific.
		Rheumatic mitral insufficiency (I05.1)	
		Mitral stenosis with insufficiency (I05.2)	
		Other mitral valve diseases (I05.8)	
		Mitral valve disease, unspecified (I05.9)	
	Rheumatic aortic valve diseases (I06)	Rheumatic aortic stenosis (I06.0)	GAS-specific.
		Rheumatic aortic insufficiency (I06.1)	
		Rheumatic aortic stenosis with insufficiency (I06.2)	
		Other rheumatic aortic valve diseases (I06.8)	
		Rheumatic aortic valve disease, unspecified (I06.9)	
	Rheumatic tricuspid valve diseases (I07)	Tricuspid stenosis (I07.0)	GAS-specific.
		Tricuspid insufficiency (I07.1)	
		Tricuspid stenosis with insufficiency (I07.2)	
		Other tricuspid valve diseases (I07.8)	
		Tricuspid valve disease, unspecified (I07.9)	
	Rheumatic disorders of both mitral and aortic valves (I08)	Disorders of both mitral and aortic valves (I08.0)	GAS-specific.
		Disorders of both mitral and tricuspid valves (I08.1)	
		Disorders of both aortic and tricuspid valves (I08.2)	
		Combined disorders of mitral, aortic and tricuspid valves (I08.3)	
		Other multiple valve diseases (I08.8)	
		Multiple valve disease, unspecified (I08.9)	
	Other rheumatic heart diseases (I09)	Rheumatic myocarditis (I09.0)	GAS-specific.
		Rheumatic diseases of endocardium, valve unspecified (I09.1)	
		Chronic rheumatic pericarditis (I09.2)	
		Other specified rheumatic heart diseases (I09.8)	
		Rheumatic heart disease, unspecified (I09.9)	

Values in brackets – International Classification of Diseases (ICD) 10 codes.

For countries where DMC values were identified through existing studies, the crude DMC was estimated for each disease by multiplying a cost per outpatient visit (and/or a cost per bed-day) obtained from the WHO-CHOICE unit cost database by the average number of outpatient visits (and/or the average length of hospital stays) which was estimated from the healthcare big data hub system. Given that the WHO-CHOICE unit costs are not GAS-specific costs, the DMC adjustment factor was generated by creating the ratio of the average of observed DMC values and the average of the crude DMC values by income group and disease. The final global DMC values for each GAS syndrome were estimated by applying the adjustment factors to the crude DMC values for all countries.

### Direct non-medical costs

Direct non-medical costs (DNMC) include costs for transportation, food, and/or lodging, etc. Similar to the DMC estimation, existing DNMC values were first extracted from literature and categorized into the diseases by income group. Considering that the average spending on DNMC is partially associated with country’s gross domestic product (GDP) per capita^29^, the observed DNMC was compared with GDP per capita for countries where DNMC were reported. In order to produce DNMC adjustment factors, the average of observed DNMC values was divided by the average of GDP per capita by income group and disease. The DNMC adjustment factors were then applied to GDP per capita for all countries to estimate DNMC values by disease. Overall, the number of data points for DNMC was smaller than for DMC. Thus, if no data was available for an income group (i.e., low-income group) or a disease, the DNMC adjustment factor was adopted from the similar income group (i.e., lower- and middle-income group) or adjusted by the duration of illness for the current disease and the similar disease category.

### Indirect costs

To estimate productivity losses due to GAS infections, extensive search was conducted to identify country’s minimum wage. While there were existing studies which reported a fairly consistent number of sick days for pharyngitis^24,30–32^, it remained challenging to identify a standardized duration of illness for other diseases due to varying degrees of subgroup definitions and categories for each disease syndrome. Thus, the aggregated number of outpatient visits and hospital bed-days was used as a proxy for the duration of illness for each of the remaining diseases based on the data obtained from the healthcare big data hub system^28^. Indirect cost (IC) was then estimated by multiplying the minimum wage by the average duration of illness by income group and disease.

### Productivity loss due to death

Productivity loss due to premature death was additionally considered. Our study focused on productivity loss due to premature death from invasive infections, RHD and severe RHD; death from pharyngitis and impetigo is extremely rare.

In order to estimate the mean age of death due to RHD, the number of deaths by age group was obtained from the Institute for Health Metrics and Evaluation (IHME)^33^, and the weighted average of age of death was calculated for all countries. In the case of invasive infections, pneumococcus was used as a proxy to estimate the weighted average of age of death based the IHME dataset. The mean age of death was then subtracted from life expectancy which was obtained from the World Bank^34^. To convert productivity loss into monetary values, productivity years lost was multiplied by minimum wage which was discounted at the rate of 3%. The final costs were expressed both in 2018 US dollars (US$) and in 2018 International dollars (I$).

### Sensitivity analysis

Given a large degree of uncertainty on input parameters, a probabilistic multivariate sensitivity analysis was extensively carried out using Ersatz^35^. A beta-PERT distribution which had been widely used for cost models was adopted for input parameters^18^. The beta-PERT distribution requires minimum, maximum, and most likely values. The function returns a random deviate from the distribution between minimum and maximum with mode^35^. The minimum and maximum values of the DMC and DNMC adjustment factors, as well as of the duration of illness for each disease category were utilized to construct the distributions. In addition, the lower- and upper-bound of the weighted average age of death were estimated based on the IHME data^33^ and used to address uncertainty surrounding productivity loss due to premature death from RHD and invasive infections. A Monte Carlo simulation was carried out based on 5000 random draws for input parameters to estimate 95% confidence intervals. Microsoft Excel 16.0.1, Ersatz 1.35, and Stata 16.1 were used for the cost estimation, sensitivity analyses, and calculating productivity loss due to premature death, respectively.

### Reporting summary

Further information on research design is available in the Nature Research Reporting Summary linked to this article.

## Supplementary information

Supplementary_clean version

REPORTING SUMMARY

## Data Availability

The datasets analyzed in the current study are in public domain and open to public users.
