# Supplementary material for: Global economic burden per episode for multiple diseases caused by group A Streptococcus
Source: NPJ Vaccines. 2023 May 15;8:69. doi: 10.1038/s41541-023-00659-1 (PMC10184078; doi:10.1038/s41541-023-00659-1)
Supplement: Supplementary file 1 — Supplementary_clean version [file 41541_2023_659_MOESM1_ESM.pdf]

Supplementary Information

Table of Contents

Existing evidence on the economic burden of GAS infections 2
Pharyngitis 2
Impetigo and Cellulitis 3
Invasive and toxin-mediated diseases 3
Acute Rheumatic Fever (ARF), Rheumatic Heart Disease (RHD), and severe RHD 4
Summary 8
References 9
Supplementary Table 2. Average number of outpatient visits and hospital bed days by disease over time 12

Existing evidence on the economic burden of GAS infections

This section describes the search outcomes of the economic burden of diseases caused by group A *Streptococcus* (GAS). The search outcomes were presented by disease manifestation: pharyngitis, impetigo, cellulitis, invasive and toxin mediated diseases, acute rheumatic fever (ARF), rheumatic heart disease (RHD), severe RHD (such as congestive heart failure, cardiac surgery, etc.). The primary focus of the search was to identify any costs associated with GAS and extract further details including healthcare descriptions, treatment items, and the unit of measure if available. More details are available at Cannon et al., “Modalities of group A streptococcal prevention and treatment and their economic justification” (currently under review by npj Vaccines).

Considering that each study reported the economic burden of a disease associated with GAS in various formats, the search outcomes were manually reviewed and categorized into direct medical cost (DMC), direct non-medical cost (DNMC), and indirect cost (IC) based on healthcare descriptions and treatment items for the purpose of the current economic burden analysis.

Pharyngitis

A total of 24 articles met the inclusion criteria and reported the three types (DMC, DNMC, or IC) of costs associated with pharyngitis or tonsillitis, or tonsillectomy. Given the limited number of data, the costs of pharyngitis and tonsillitis were considered to be in the same disease group.

Supplementary Table 1.1 shows the number of studies from which cost data were extracted for pharyngitis and tonsillectomy. As indicated in the table, a majority of the costs were observed in high-income country settings. Regarding the cost type, it appeared that DMC was the most commonly reported form followed by IC and DNMC. The number of studies was significantly lower in other income settings than in the high-income group, and there was no study available for the low-income group. In fact, Manji et al. targeted developing countries for their cost-effectiveness analysis mentioning sub-Saharan African in their paper [1], but this study was excluded because the source of costing data was not clearly indicated, and they reported annual costs rather than cost per episode or cost per patient. The economic burden of tonsillectomy was only available from high-income country settings.

Supplementary Table 1.1 Number of studies for pharyngitis

Income group	Cost type	Disease type	Source
		Pharyngitis (or tonsillitis)	
High income	DMC	21	[2-22]
	DNMC	2	[3, 20]
	DMC&DNMC	-	
	IC	5	[3, 6, 15, 20, 22]
Upper-middle income	DMC	2	[23, 24]
	DNMC	-	
	DMC&DNMC	-	
	IC	-	
	DMC	1	[25]

Lower-middle income	DNMC	1	[25]
	DMC&DNMC	-	
	IC	-	
Low income	DMC	-	
	DNMC	-	
	DMC&DNMC	-	
	IC	-	

Impetigo and Cellulitis

For GAS skin-related infections such as impetigo, and cellulitis, there was clearly a lack of existing studies which report economic burden. As shown in Supplementary Table 1.2, there were no studies identified for the skin infections in non-high-income settings except one for cellulitis in the upper-middle income group and the other for impetigo in the lower-middle income group. In particular, the economic burden of impetigo was rare even in the high-income settings showing only one study of each.

Supplementary Table 1.2 Number of studies for impetigo and cellulitis

Income group	Cost type	Disease type		Source
		Impetigo	Cellulitis	
High income	DMC	1	9	[2, 26-33]
	DNMC	-	-	
	DMC&DNMC	-	-	
	IC	-	2	[26, 28]
Upper-middle income	DMC	-	1	[34]
	DNMC	-	-	
	DMC&DNMC	-	-	
	IC	-	-	
Lower-middle income	DMC	1	-	[35]
	DNMC	-	-	
	DMC&DNMC	-	-	
	IC	-	-	
Low income	DMC	-	-	
	DNMC	-	-	
	DMC&DNMC	-	-	
	IC	-	-	

Invasive and toxin-mediated diseases

GAS sometimes develops into invasive or toxin-mediated diseases Supplementary Table 1.3 demonstrates the number of studies which reports the economic burden of each infection. Similar to GAS skin

infections, while the occurrence of such infections has been reported in existing literature, the number of studies which reports the economic burden of them is very limited.

Supplementary Table 1.3 Number of studies for invasive and toxin mediated diseases

Income group	Cost type	Disease type	Source
		Invasive and toxin-mediated	
High income	DMC	5	[2, 36-39]
	DNMC	-	
	DMC&DNMC	-	
	IC	-	
Upper-middle income	DMC	-	
	DNMC	-	
	DMC&DNMC	-	
	IC	-	
Lower-middle income	DMC	-	
	DNMC	-	
	DMC&DNMC	-	
	IC	-	
Low income	DMC	-	
	DNMC	-	
	DMC&DNMC	-	
	IC	-	

Acute Rheumatic Fever (ARF), Rheumatic Heart Disease (RHD), and severe RHD

There were 18 studies which reported the economic burden of either acute rheumatic fever (ARF), rheumatic heart disease (RHD), or severe RHD (or any combination of these). Severe RHD was defined if a patient went through any of the following processes: (congestive) heart failure, stroke, or any surgery-related procedures due to RHD. Overall, a smaller number of studies were identified compared to pharyngitis. As shown in Supplementary Table 1.4, The availability of the cost data for ARF, RHD, or severe RHD showed the similar patterns described for pharyngitis: most of the studies coming from the high-income countries, and DMC being the most commonly reported form of the economic burden. Again, no study was identified in low-income group settings, and there were only three and four studies identified in the upper-middle income and the lower-middle income settings, respectively. Among the studies which reported DMC in the lower-middle income group, one of them constructed a cost-effectiveness model targeting the African region [40], but their costs were mainly taken from a previous study conducted in Cuba [41] which belongs to the upper-middle income group. Given the previous study done in Cuba was already included in the upper-middle income group, the costs used in the other study [40] were not included to avoid any duplication. Soudarssanane et al. reported multiple cost items in three categories [25]: primary prevention, secondary prevention, and tertiary prevention. For the purpose of the current screening process, each cost of primary, secondary, and tertiary prevention was assumed to be that of ARF, RHD, and severe RHD respectively. In addition, the cost for ARF or mild RHD was grouped

into the cost for ARF, and the cost of moderate RHD was considered to be the cost of RHD for the study done by Cannon et al. [2].

Supplementary Table 1.4 Number of studies for ARF, RHD, and severe RHD

Income group	Cost type	Disease type			Source
		ARF	RHD	severe RHD	
High income	DMC	8	7	4	[3, 4, 10, 12, 14-16, 19, 22, 42-44]
	DNMC	-	3	2	[3, 4, 42]
	DMC&DNMC	2	2	2	[2] (general and indigenous populations separately)
	IC	2	3	1	[3, 15, 22, 42]
Upper-middle income	DMC	2	2	2	[41, 45]
	DNMC	-	-	-	
	DMC&DNMC	1	-	-	[46]
	IC	1	-	-	[46]
Lower-middle income	DMC	1	2	2	[25, 47]
	DNMC	1	1	-	[25]
	DMC&DNMC	-	-	-	
	IC	1	1	-	[25]
Low income	DMC	-	-	-	
	DNMC	-	-	-	
	DMC&DNMC	-	-	-	
	IC	-	-	-	

Supplementary Table 1.5 Direct medical costs and direct non-medical costs extracted from existing literature*

Income group	Pharyngitis			Impetigo			Cellulitis			Invasive and toxin-mediated infections			ARF			RHD			Severe RHD		
	Direct Medical Costs (DMC)			Direct Medical Costs (DMC)			Direct Medical Costs (DMC)			Direct Medical Costs (DMC)			Direct Medical Costs (DMC)			Direct Medical Costs (DMC)			Direct Medical Costs (DMC)		
**High income**	Australia	Cannon, 2018	295.10	Australia	Cannon, 2018	3630.89	Australia	Cannon, 2018	4475.98	Australia	Cannon, 2018	18191.30	New Zealand	Oetzel 2019	110.27	Australia	Roberts 2017	8403.89	Australia	Roberts 2017	77227.81
	Australia	Zachariah 2015	18.85				Australia	Ibrahim 2019	2873.94	Australia	Widjaja, 2005	74912.48	Spain	Giraldez-Garcia 2011	3,023.81	Australia	Zachariah 2015	569.03	Australia	Zachariah 2015	19542.05
	Australia	Roberts 2017	68.18				Australia	Kameshwar 2016	4114.38	United Kingdom	Hughes, 2015	11132.12	United Kingdom	Little 2014	4,888.25	Korea, Rep.	Seo 2013	409.90	United Kingdom	Little 2014	16837.13
	Bahrain	Al Alawi 2015	96.57				United Kingdom	Brugha 2012	6187.33	United States	Al Alawi 2015	75518.76	United States	Ehrlich 2002	8,681.03	New Zealand	Milne 2012	3555.67	United States	King 2002	86810.31
	Belgium	Leupe 2012	53.47				United Kingdom	Mason 2014	1062.99	United States	Leupe 2012	39065.58	United States	King 2002	15,689.52	United Kingdom	Little 2014	3632.41			
	France	Maizia 2012 (French)	46.29				United States	Kim 2012	1223.40				United States	Klepser 2011	3,248.93	United States	King 2002	26524.89			
	Japan	Fujihara 2006	80.87				United States	Li 2018	335.32				United States	Neuner 2003	2,666.21	United States	Van Howe 2006	26768.15			
	Poland	Bura 2017	8.87				United States	Peterson 2017	5602.15				United States	Van Howe 2006	8,030.45						
	Spain	Giraldez-Garcia 2011	72.07				United States	St John 2018	625.49												
	Switzerland	Humair 2006	51.63																		
	United Kingdom	Little 2014	233.73																		
	United States	Bhattacharyya 2002	64.96																		
	United States	Ehrlich 2002	86.81																		
	United States	King 2002	193.88																		
	United States	Klepser 2011	227.62																		
	United States	Mayes 2001	19.87																		
	United States	Nakhoul 2013	131.43																		
	United States	Neuner 2003	32.72																		

	United States; Pfoh 2008; 169.30<br>United States; Salkind 2008; 149.67<br>United States; Van Howe 2006; 79.55						
	**Direct Non-Medical Costs (DNMC)**	**Direct Non-Medical Costs (DNMC)**	**Direct Non-Medical Costs (DNMC)**	**Direct Non-Medical Costs (DNMC)**	**Direct Non-Medical Costs (DNMC)**	**Direct Non-Medical Costs (DNMC)**	**Direct Non-Medical Costs (DNMC)**
	Australia; Zachariah 2015; 8.57<br>United States; Pfoh 2008; 15.95	*No data available*	*No data available*	*No data available*	*No data available*	Australia; Roberts 2017; 4292.33<br>Australia; Zachariah 2015; 8.57<br>Korea, Rep.; Seo 2013; 50.27	Australia; Roberts 2017; 3199.82<br>Australia; Zachariah 2015; 248.09
**Upper-Middle income**	**Direct Medical Costs (DMC)**	**Direct Medical Costs (DMC)**	**Direct Medical Costs (DMC)**	**Direct Medical Costs (DMC)**	**Direct Medical Costs (DMC)**	**Direct Medical Costs (DMC)**	**Direct Medical Costs (DMC)**
	South Africa; Irlam 2013; 33.58<br>Turkey; Kose 2016; 14.68	*No data available*	Turkey; Caglar 2018; 283.31	*No data available*	Cuba; Watkins 2015; 2233.21<br>South Africa; Irlam 2013; 4935.06	Cuba; Watkins 2015; 3364.14<br>South Africa; Irlam 2013; 3667.20	Cuba; Watkins 2015; 8208.54<br>South Africa; Irlam 2013; 16998.37
	**Direct Non-Medical Costs (DNMC)**	**Direct Non-Medical Costs (DNMC)**	**Direct Non-Medical Costs (DNMC)**	**Direct Non-Medical Costs (DNMC)**	**Direct Non-Medical Costs (DNMC)**	**Direct Non-Medical Costs (DNMC)**	**Direct Non-Medical Costs (DNMC)**
	No data available	*No data available*	*No data available*	*No data available*	*No data available*	*No data available*	*No data available*
**Lower-Middle income**	**Direct Medical Costs (DMC)**	**Direct Medical Costs (DMC)**	**Direct Medical Costs (DMC)**	**Direct Medical Costs (DMC)**	**Direct Medical Costs (DMC)**	**Direct Medical Costs (DMC)**	**Direct Medical Costs (DMC)**
	India; Soudarssanane 2007; 4.05	India; Rani 2019; 1.76	*No data available*	*No data available*	India; Soudarssanane 2007; 230.24	India; Soudarssanane 2007; 804.17<br>Kenya; Kontchou 2019; 163.87	India; Soudarssanane 2007; 2163.84<br>Kenya; Kontchou 2019; 237.08
	**Direct Non-Medical Costs (DNMC)**	**Direct Non-Medical Costs (DNMC)**	**Direct Non-Medical Costs (DNMC)**	**Direct Non-Medical Costs (DNMC)**	**Direct Non-Medical Costs (DNMC)**	**Direct Non-Medical Costs (DNMC)**	**Direct Non-Medical Costs (DNMC)**
	India; Soudarssanane 2007; 0.72	*No data available*	*No data available*	*No data available*	India; Soudarssanane 2007; 0.95	India; Soudarssanane 2007; 12.40	*No data available*
**Low income**	**Direct Medical Costs (DMC)**	**Direct Medical Costs (DMC)**	**Direct Medical Costs (DMC)**	**Direct Medical Costs (DMC)**	**Direct Medical Costs (DMC)**	**Direct Medical Costs (DMC)**	**Direct Medical Costs (DMC)**
	No data available	*No data available*	*No data available*	*No data available*	*No data available*	*No data available*	*No data available*
	**Direct Non-Medical Costs (DNMC)**	**Direct Non-Medical Costs (DNMC)**	**Direct Non-Medical Costs (DNMC)**	**Direct Non-Medical Costs (DNMC)**	**Direct Non-Medical Costs (DNMC)**	**Direct Non-Medical Costs (DNMC)**	**Direct Non-Medical Costs (DNMC)**
	No data available	*No data available*	*No data available*	*No data available*	*No data available*	*No data available*	*No data available*

[*] The values are inflated and converted into USD as described in the main text.

Summary

The economic burden of GAS infections is scarce in existing literature. Among a few studies available, most of them reported the costs from the same high-income countries such as Australia, and United States. The number of available studies was disproportionately lower in upper-middle-, lower-middle-, and low-income countries. In particular, there was no study identified in the low-income settings for any of the GAS-associated manifestations. This is partly because a majority of the costs data identified in this search were taken from existing cost-effectiveness analyses which had been mainly conducted in the high-income country settings. This highlights the significant gap in health economic studies including cost of illness, particularly in non-high income settings.

References

1. Manji RA, Witt J, Tappia PS, Jung Y, Menkis AH, Ramjiawan B: **Cost-effectiveness analysis of rheumatic heart disease prevention strategies**. *Expert review of pharmacoeconomics & outcomes research* 2013, **13**(6):715-724.
2. Cannon JW, Jack S, Wu Y, Zhang J, Baker MG, Geelhoed E, Fraser J, Carapetis JR: **An economic case for a vaccine to prevent group A streptococcus skin infections**. *Vaccine* 2018, **36**(46):6968-6978.
3. Zachariah JP, Samnaliev M: **Echo-based screening of rheumatic heart disease in children: a cost-effectiveness Markov model**. *J Med Econ* 2015, **18**(6):410-419.
4. Roberts K, Cannon J, Atkinson D, Brown A, Maguire G, Remenyi B, Wheaton G, Geelhoed E, Carapetis JR: **Echocardiographic Screening for Rheumatic Heart Disease in Indigenous Australian Children: A Cost-Utility Analysis**. *J Am Heart Assoc* 2017, **6**(3).
5. Al Alawi S, Abdulkarim S, Elhennawy H, Al-Mansoor A, Al Ansari A: **Outpatient parenteral antimicrobial therapy with ceftriaxone for acute tonsillopharyngitis: efficacy, patient satisfaction, cost effectiveness, and safety**. *Infect Drug Resist* 2015, **8**:279-285.
6. Leupe P, Hox V, Debruyne F, Schrooten W, Claes NV, Lemkens N, Lemkens P: **Tonsillectomy compared to acute tonsillitis in children: a comparison study of societal costs**. *B-ent* 2012, **8**(2):103-111.
7. Maizia A, Letrilliart L, Colin C: **[Diagnostic strategies for acute tonsillitis in France: a cost-effectiveness study]**. *Presse Med* 2012, **41**(4):e195-203.
8. Fujihara K, Koltai PJ, Hayashi M, Tamura S, Yamanaka N: **Cost-Effectiveness of Tonsillectomy for Recurrent Acute Tonsillitis**. *Annals of Otology, Rhinology & Laryngology* 2006, **115**(5):365-369.
9. Bura M, Michalak M, Chojnicki M, Padzik M, Mozer-Lisewska I: **Moderate and severe pharyngitis in young adult inhabitants of Poznan, western Poland**. *Family Medicine & Primary Care Review* 2017, **1**:12-17.
10. Giraldez-Garcia C, Rubio B, Gallegos-Braun JF, Imaz I, Gonzalez-Enriquez J, Sarria-Santamera A: **Diagnosis and management of acute pharyngitis in a paediatric population: a cost-effectiveness analysis**. *Eur J Pediatr* 2011, **170**(8):1059-1067.
11. Humair JP, Revaz SA, Bovier P, Stalder H: **Management of acute pharyngitis in adults: reliability of rapid streptococcal tests and clinical findings**. *Arch Intern Med* 2006, **166**(6):640-644.
12. Little P, Hobbs FD, Moore M, Mant D, Williamson I, McNulty C, Lasseter G, Cheng MY, Leydon G, McDermott L *et al*: **PRImary care Streptococcal Management (PRISM) study: in vitro study, diagnostic cohorts and a pragmatic adaptive randomised controlled trial with nested qualitative study and cost-effectiveness study**. *Health Technol Assess* 2014, **18**(6):vii-xxv, 1-101.
13. Bhattacharyya N, Kepnes LJ: **Economic benefit of tonsillectomy in adults with chronic tonsillitis**. *The Annals of otology, rhinology, and laryngology* 2002, **111**(11):983-988.
14. Ehrlich JE, Demopoulos BP, Daniel KR, Jr., Ricarte MC, Glied S: **Cost-effectiveness of treatment options for prevention of rheumatic heart disease from Group A streptococcal pharyngitis in a pediatric population**. *Prev Med* 2002, **35**(3):250-257.
15. King CH, Fischler DF, Gerkin RD: **Will genetic testing alter the management of disease caused by infectious agents? A cost-effectiveness analysis of gene-testing strategies for prevention of rheumatic Fever**. *Clin Infect Dis* 2002, **34**(11):1491-1499.
16. Klepser DG, Bisanz SE, Klepser ME: **Cost-effectiveness of pharmacist-provided treatment of adult pharyngitis**. *The American journal of managed care* 2012, **18**(4):e145-154.

17. Mayes T, Pichichero ME: **Are follow-up throat cultures necessary when rapid antigen detection tests are negative for group A streptococci?** *Clin Pediatr (Phila)* 2001, **40**(4):191-195.
18. Nakhoul GN, Hickner J: **Management of adults with acute streptococcal pharyngitis: minimal value for backup strep testing and overuse of antibiotics**. *J Gen Intern Med* 2013, **28**(6):830-834.
19. Neuner JM, Hamel MB, Phillips RS, Bona K, Aronson MD: **Diagnosis and management of adults with pharyngitis. A cost-effectiveness analysis**. *Ann Intern Med* 2003, **139**(2):113-122.
20. Pfoh E, Wessels MR, Goldmann D, Lee GM: **Burden and economic cost of group A streptococcal pharyngitis**. *Pediatrics* 2008, **121**(2):229-234.
21. Salkind AR, Wright JM: **Economic burden of adult pharyngitis: the payer's perspective**. *Value Health* 2008, **11**(4):621-627.
22. Van Howe RS, Kusnier LP, 2nd: **Diagnosis and management of pharyngitis in a pediatric population based on cost-effectiveness and projected health outcomes**. *Pediatrics* 2006, **117**(3):609-619.
23. Irlam J, Mayosi BM, Engel M, Gaziano TA: **Primary prevention of acute rheumatic fever and rheumatic heart disease with penicillin in South African children with pharyngitis: a cost-effectiveness analysis**. *Circ Cardiovasc Qual Outcomes* 2013, **6**(3):343-351.
24. Kose E, Sirin Kose S, Akca D, Yildiz K, Elmas C, Baris M, Anil M: **The Effect of Rapid Antigen Detection Test on Antibiotic Prescription Decision of Clinicians and Reducing Antibiotic Costs in Children with Acute Pharyngitis**. *J Trop Pediatr* 2016, **62**(4):308-315.
25. Soudarssanane MB, Karthigeyan M, Mahalakshmy T, Sahai A, Srinivasan S, Subba Rao KS, Balachander J: **Rheumatic fever and rheumatic heart disease: primary prevention is the cost effective option**. *Indian journal of pediatrics* 2007, **74**(6):567-570.
26. Ibrahim LF, Huang L, Hopper SM, Dalziel K, Babl FE, Bryant PA: **Intravenous ceftriaxone at home versus intravenous flucloxacillin in hospital for children with cellulitis: a cost-effectiveness analysis**. *The Lancet Infectious Diseases* 2019, **19**(10):1101-1108.
27. Kameshwar K, Karahalios A, Janus E, Karunajeewa H: **False economies in home-based parenteral antibiotic treatment: a health-economic case study of management of lower-limb cellulitis in Australia**. *J Antimicrob Chemother* 2016, **71**(3):830-835.
28. Mason JM, Thomas KS, Crook AM, Foster KA, Chalmers JR, Nunn AJ, Williams HC: **Prophylactic antibiotics to prevent cellulitis of the leg: economic analysis of the PATCH I & II trials**. *PloS one* 2014, **9**(2):e82694.
29. Li DG, Xia FD, Khosravi H, Dewan AK, Pallin DJ, Baugh CW, Laskowski K, Joyce C, Mostaghimi A: **Outcomes of Early Dermatology Consultation for Inpatients Diagnosed With Cellulitis**. *JAMA Dermatol* 2018, **154**(5):537-543.
30. Peterson RA, Polgreen LA, Cavanaugh JE, Polgreen PM: **Increasing Incidence, Cost, and Seasonality in Patients Hospitalized for Cellulitis**. *Open Forum Infect Dis* 2017, **4**(1):ofx008.
31. St John J, Strazzula L, Vedak P, Kroshinsky D: **Estimating the health care costs associated with recurrent cellulitis managed in the outpatient setting**. *J Am Acad Dermatol* 2018, **78**(4):749-753.
32. Brugha RE, Abrahamson E: **Ambulatory Intravenous Antibiotic Therapy for Children With Preseptal Cellulitis**. *Pediatric Emergency Care* 2012, **28**(3):226-228.
33. Kim MK, Allareddy V, Nalliah RP, Kim JE, Allareddy V: **Burden of facial cellulitis: estimates from the Nationwide Emergency Department Sample**. *Oral Surgery Oral Medicine Oral Pathology Oral Radiology* 2012, **114**(3):312-317.

34. Çağlar İ, Kafes C, Korcum M, Düzgöl M, Kara A, Bayram SN, Apa H, Devrim İ: **Hospital cost analysis of children with preseptal cellulitis**. *International Journal of Pediatric Otorhinolaryngology* 2018, **106**:96-99.
35. Anusha Rani MV, Bhuvaneshwari E, Venkatakrishna A: **Comparison of efficacy and cost-effectiveness of topical fusidic acid and topical mupirocin in the treatment of impetigo**. *National Journal of Physiology, Pharmacy and Pharmacology* 2019, **9**(12):1225-1229.
36. Widjaja AB, Tran A, Cleland H, Leung M, Millar I: **The hospital costs of treating necrotizing fasciitis**. *Anz Journal of Surgery* 2005, **75**(12):1059-1064.
37. Hughes GJ, Van Hoek AJ, Sriskandan S, Lamagni TL: **The cost of hospital care for management of invasive group A streptococcal infections in England**. *Epidemiology and Infection* 2015, **143**(8):1719-1730.
38. Mulla ZD, Gibbs SG, Aronoff DM: **Correlates of length of stay, cost of care, and mortality among patients hospitalized for necrotizing fasciitis**. *Epidemiology and Infection* 2007, **135**(5):868-876.
39. Strom MA, Hsu DY, Silverberg JI: **Prevalence, comorbidities and mortality of toxic shock syndrome in children and adults in the USA**. *Microbiology and Immunology* 2017, **61**(11):463-473.
40. Watkins D, Lubinga SJ, Mayosi B, Babigumira JB: **A Cost-Effectiveness Tool to Guide the Prioritization of Interventions for Rheumatic Fever and Rheumatic Heart Disease Control in African Nations**. *PLoS Negl Trop Dis* 2016, **10**(8):e0004860.
41. Watkins DA, Mvundura M, Nordet P, Mayosi BM: **A cost-effectiveness analysis of a program to control rheumatic fever and rheumatic heart disease in Pinar del Rio, Cuba**. *PloS one* 2015, **10**(3):e0121363.
42. Seo HY, Yoon SJ, Kim EJ, Oh IH, Lee YH, Kim YA: **The economic burden of rheumatic heart disease in South Korea**. *Rheumatol Int* 2013, **33**(6):1505-1510.
43. Milne RJ, Lennon D, Stewart JM, Vander Hoorn S, Scuffham PA: **Mortality and hospitalisation costs of rheumatic fever and rheumatic heart disease in New Zealand**. *J Paediatr Child Health* 2012, **48**(8):692-697.
44. Oetzel JG, Lao C, Morley M, Penman K, Child M, Scott N, Karalus M: **Efficacy of an incentive intervention on secondary prophylaxis for young people with rheumatic fever: a multiple baseline study**. *BMC Public Health* 2019, **19**(1):385.
45. Irlam JH, Mayosi BM, Engel ME, Gaziano TA: **A cost-effective strategy for primary prevention of acute rheumatic fever and rheumatic heart disease in children with pharyngitis**. *S Afr Med J* 2013, **103**(12):894-895.
46. Terreri MT, Ferraz MB, Goldenberg J, Len C, Hilário MO: **Resource utilization and cost of rheumatic fever**. *J Rheumatol* 2001, **28**(6):1394-1397.
47. Kontchou NT, McCrary AW, Schulman KA: **Workforce Cost Model for Expanding Congenital and Rheumatic Heart Disease Services in Kenya**. *World J Pediatr Congenit Heart Surg* 2019, **10**(3):321-327.

Supplementary Table 2. Average number of outpatient visits and hospital bed days by disease over time

Disease	Type	Year									
		**2010**	**2011**	**2012**	**2013**	**2014**	**2015**	**2016**	**2017**	**2018**	**2019**
Pharyngitis	OP visits	1.9	1.8	1.9	1.8	1.8	1.8	1.8	1.7	1.7	1.7
	IP bed days	5.1	4.9	4.8	4.7	4.8	4.3	4.5	4.3	3.7	3.9
Impetigo	OP visits	1.5	1.6	1.6	1.8	1.6	1.7	1.7	1.6	1.6	1.6
	IP bed days	6.8	17.0	2.0	0.0	0.0	0.0	0.0	3.0	0.0	0.0
Cellulitis	OP visits	2.5	3.1	3.3	2.8	1.9	1.7	1.7	1.6	1.6	1.7
	IP bed days	12.3	10.4	8.9	9.0	8.8	6.0	12.0	4.2	6.0	0.0
Invasive infections	OP visits	2.8	2.5	2.4	2.4	2.4	2.4	2.4	2.3	2.4	2.6
	IP bed days	21.8	18.9	19.1	17.4	17.5	19.3	18.7	14.2	16.1	18.5
ARF	OP visits	2.2	2.4	3.6	3.9	4.6	4.9	4.2	4.2	3.6	3.0
	IP bed days	12.4	9.7	13.1	17.1	29.8	29.5	14.0	18.6	17.4	16.1
RHD	OP visits	4.4	2.8	3.0	2.7	2.8	2.5	2.2	2.3	2.0	2.3
	IP bed days	10.9	16.6	54.8	12.1	16.1	7.0	20.5	46.0	14.2	5.0
Severe RHD	OP visits	4.4	4.3	4.4	4.2	4.3	4.2	4.1	4.0	4.2	4.0
	IP bed days	17.8	17.1	17.0	16.5	16.4	16.9	16.6	17.0	18.1	17.5
