# Supplementary material for: Global economic burden per episode for multiple diseases caused by group A Streptococcus
Source: NPJ Vaccines. 2023 May 15;8:69. doi: 10.1038/s41541-023-00659-1 (PMC10184078; doi:10.1038/s41541-023-00659-1)
Supplement: Supplementary file 2 — REPORTING SUMMARY [file 41541_2023_659_MOESM2_ESM.pdf]

nature portfolio

Corresponding author(s): Jung-Seok Lee
Last updated by author(s): Oct 11, 2022

Reporting Summary

Nature Portfolio wishes to improve the reproducibility of the work that we publish. This form provides structure for consistency and transparency in reporting. For further information on Nature Portfolio policies, see our Editorial Policies and the Editorial Policy Checklist.

Statistics

For all statistical analyses, confirm that the following items are present in the figure legend, table legend, main text, or Methods section.

n/a	Confirmed	
☒	☐	The exact sample size (n) for each experimental group/condition, given as a discrete number and unit of measurement
☒	☐	A statement on whether measurements were taken from distinct samples or whether the same sample was measured repeatedly
☒	☐	The statistical test(s) used AND whether they are one- or two-sided<br>*Only common tests should be described solely by name; describe more complex techniques in the Methods section.*
☒	☐	A description of all covariates tested
☐	☒	A description of any assumptions or corrections, such as tests of normality and adjustment for multiple comparisons
☐	☒	A full description of the statistical parameters including central tendency (e.g. means) or other basic estimates (e.g. regression coefficient) AND variation (e.g. standard deviation) or associated estimates of uncertainty (e.g. confidence intervals)
☒	☐	For null hypothesis testing, the test statistic (e.g. F, t, r) with confidence intervals, effect sizes, degrees of freedom and P value noted<br>*Give P values as exact values whenever suitable.*
☒	☐	For Bayesian analysis, information on the choice of priors and Markov chain Monte Carlo settings
☒	☐	For hierarchical and complex designs, identification of the appropriate level for tests and full reporting of outcomes
☒	☐	Estimates of effect sizes (e.g. Cohen's d, Pearson's r), indicating how they were calculated

Our web collection on statistics for biologists contains articles on many of the points above.

Software and code

Policy information about availability of computer code

Data collection	Data were collected from existing literature, WHO-CHOICE (open dataset), and Healthcare Big data Hub (open dataset).
Data analysis	Microsoft Excel, Ersatz, and STATA were used for the cost estimation, sensitivity analyses, and calculating productivity loss due to premature death, respectively.

For manuscripts utilizing custom algorithms or software that are central to the research but not yet described in published literature, software must be made available to editors and reviewers. We strongly encourage code deposition in a community repository (e.g. GitHub). See the Nature Portfolio guidelines for submitting code & software for further information.

Data

Policy information about availability of data

All manuscripts must include a data availability statement. This statement should provide the following information, where applicable:

- Accession codes, unique identifiers, or web links for publicly available datasets
- A description of any restrictions on data availability
- For clinical datasets or third party data, please ensure that the statement adheres to our policy

The datasets analyzed in the current study are in public domain and open to public users.

Human research participants

Policy information about studies involving human research participants and Sex and Gender in Research.

Reporting on sex and gender	NA
Population characteristics	NA
Recruitment	The study did not recruit any participants.
Ethics oversight	The study did not recruit any participants.

Note that full information on the approval of the study protocol must also be provided in the manuscript.

Field-specific reporting

Please select the one below that is the best fit for your research. If you are not sure, read the appropriate sections before making your selection.

☐ Life sciences ☒ Behavioural & social sciences ☐ Ecological, evolutionary & environmental sciences

For a reference copy of the document with all sections, see nature.com/documents/nr-reporting-summary-flat.pdf

Behavioural & social sciences study design

All studies must disclose on these points even when the disclosure is negative.

Study description	The study is the estimation of economic burden for GAS infections using data from existing literature and public data sources.
Research sample	Data were collected from existing literature and public data sources.
Sampling strategy	We included all relevant data points identified in existing literature. Sample-size calculation was not performed as the aim of the current study was to extrapolate values by income group based on adjustment factors, rather than conducting statistical analyses.
Data collection	Microsoft Excel was used for data collection. Two authors (JSL and SK) cross-checked the dataset for the relevance of data to the analysis.
Timing	Data collection was performed between Jun 2020 to Mar 2021.
Data exclusions	From the literature searched, four studies were excluded: (1) one of them did not clearly indicate the source of costing data, (2) one used the same costing data as another study, (3) two of them did not report separate estimates for DMC and DNMC.
Non-participation	The study did not involve any participants.
Randomization	The study did not involve any participants.

Reporting for specific materials, systems and methods

We require information from authors about some types of materials, experimental systems and methods used in many studies. Here, indicate whether each material, system or method listed is relevant to your study. If you are not sure if a list item applies to your research, read the appropriate section before selecting a response.

Materials & experimental systems

n/a	Involved in the study	
☒	☐	Antibodies
☒	☐	Eukaryotic cell lines
☒	☐	Palaeontology and archaeology
☒	☐	Animals and other organisms
☒	☐	Clinical data
☒	☐	Dual use research of concern

Methods

n/a	Involved in the study	
☒	☐	ChIP-seq
☒	☐	Flow cytometry
☒	☐	MRI-based neuroimaging
